# Every Night and Every Morn: Effect of Variation in *CLOCK* Gene on Depression Depends on Exposure to Early and Recent Stress

**DOI:** 10.3389/fpsyt.2021.687487

**Published:** 2021-08-26

**Authors:** Dorka Gyorik, Nora Eszlari, Zsofia Gal, Dora Torok, Daniel Baksa, Zsuliet Kristof, Sara Sutori, Peter Petschner, Gabriella Juhasz, Gyorgy Bagdy, Xenia Gonda

**Affiliations:** ^1^Faculty of Medicine, Semmelweis University, Budapest, Hungary; ^2^Department of Pharmacodynamics, Faculty of Pharmacy, Semmelweis University, Budapest, Hungary; ^3^NAP-2-SE New Antidepressant Target Research Group, Hungarian Brain Research Program, Semmelweis University, Budapest, Hungary; ^4^SE-NAP-2 Genetic Brain Imaging Migraine Research Group, Hungarian Brain Research Program, Semmelweis University, Budapest, Hungary; ^5^Doctoral School of Mental Health Sciences, Semmelweis University, Budapest, Hungary; ^6^Laboratory of Molecular Pharmacology, Institute of Experimental Medicine, Hungarian Academy of Sciences, Budapest, Hungary; ^7^Department of Psychiatry and Psychotherapy, Semmelweis University, Budapest, Hungary; ^8^MTA-SE Neuropsychopharmacology and Neurochemistry Research Group, Hungarian Academy of Sciences, Semmelweis University, Budapest, Hungary

**Keywords:** clock gene, depression, stress, childhood adversities, negative life events, gene-environment interactions, circadian rhythm

## Abstract

The role of circadian dysregulation is increasingly acknowledged in the background of depressive symptoms, and is also a promising treatment target. Similarly, stress shows a complex relationship with the circadian system. The *CLOCK* gene, encoding a key element in circadian regulation has been implicated in previous candidate variant studies in depression with contradictory findings, and only a few such studies considered the interacting effects of stress. We investigated the effect of *CLOCK* variation with a linkage-disequilibrium-based clumping method, in interaction with childhood adversities and recent negative life events, on two phenotypes of depression, lifetime depression and current depressive symptoms in a general population sample.

**Methods:** Participants in NewMood study completed questionnaires assessing childhood adversities and recent negative life events, the Brief Symptom Inventory to assess current depressive symptoms, provided data on lifetime depression, and were genotyped for 1054 SNPs in the *CLOCK* gene, 370 of which survived quality control and were entered into linear and logistic regression models with current depressive symptoms and lifetime depression as the outcome variable, and childhood adversities or recent life events as interaction variables followed by a linkage disequilibrium-based clumping process to identify clumps of SNPs with a significant main or interaction effect.

**Results:** No significant clumps with a main effect were found. In interaction with recent life events a significant clump containing 94 SNPs with top SNP rs6825994 for dominant and rs6850524 for additive models on current depression was identified, while in interaction with childhood adversities on current depressive symptoms, two clumps, both containing 9 SNPs were found with top SNPs rs6828454 and rs711533.

**Conclusion:** Our findings suggest that *CLOCK* contributes to depressive symptoms, but via mediating the effects of early adversities and recent stressors. Given the increasing burden on circadian rhythmicity in the modern lifestyle and our expanding insight into the contribution of circadian disruption in depression especially as a possible mediator of stress, our results may pave the way for identifying those who would be at an increased risk for depressogenic effects of circadian dysregulation in association with stress as well as new molecular targets for intervention in stress-related psychopathologies in mood disorders.

## Introduction

In order to successfully adapt to environmental changes including day-night cycles signaling rhythmic alterations in the availability of resources and presence of dangers, regulation of rhythmic metabolic, cognitive, and behavioral functions via optimization of physiological and biological processes to 24-h cycles is necessary (1). Circadian disruption may compromise survival and lead to the emergence of several somatic and mental disorders including depression (2, 3). Self-sustained biological rhythms are controlled by the circadian system composed of tissues expressing endogenous 24-h timekeeping activity (4). The nucleus suprachiasmaticus acts as central pacemaker, the activity of which is based on a transcriptional/posttranslational feedback loop with rhythmic expression of circadian clock genes (5), including the *CLOCK* gene (circadian locomotor output cycles kaput gene) which possesses a transcriptional activator role in the circadian clock mechanism.

Genetic mutations in clock genes may lead to disruptions in the period, phase and amplitude of circadian rhythms (6). In animal models, manipulation and mutation of clock genes causes alterations in behavioral and affective phenotypes, suggesting a direct connection between clock genes and brain functions relevant to psychiatric illness (7). Depression has been linked to circadian abnormalities for decades as suggested by somatic rhythms showing disruption in depressed patients, as well as a typical circadian rhythmicity of various symptoms (8). Prominent circadian disturbances in mood disorders include sleep problems, morning worsening and evening improvement of symptoms, changes in appetite, social interactions, as well as alteration in the circadian rhythmicity of blood pressure, body temperature or hormone levels (9–11). These have implicated a rhythm disruption of central origin involving the core molecular machinery underlying circadian rhythm generation, thus suggesting that disruption of circadian regulation is a factor possibly underlying the development and maintenance of the disorder (12–14).

There is a strong genetic background of mood disorders (15, 16) and some studies point to an association between *CLOCK* gene variation and bipolar disorder (17, 18) but weak association with major depressive disorder (MDD) (12, 17, 19). However, in case of depression, the heterogeneity of the disorder and the underlying neurobiological etiological processes raised the possibility that inaccurate or misinterpreted phenotypes (19) and lack of differentiation between depression subtypes may have obscured existing associations. Furthermore, the circadian clock and stress response systems are closely related (7), and stress/increased vulnerability to stress are risk factors for multiple psychiatric disorders. Modulation of stress response is a common mechanism by which circadian clock genes affect such illnesses including depression (7). It has been suggested that effects of SNPs in candidate gene studies and GWAS-s may be masked by lack of consideration of the interacting effects of various types of environmental events in spite of our increasing understanding that the majority of genes and variants contributing to the emergence of depression act via modulating sensitivity toward stress (16, 20, 21).

In previous studies, only a few candidate polymorphisms in the *CLOCK* gene, and most frequently rs1801260 (also known as 3111T/C) were investigated in association with depression with inconsistent findings. GWAS-s have not confirmed the role of this variant or the *CLOCK* gene, although suggested a role for circadian system genes (19, 22, 23). However, GWAS-s may overlook existing association due to strict *p*-value criteria to compensate for multiple testing with even true positive SNPs not achieving genome-wide significance (19, 24). One way of reducing multiple testing burden yet overcoming the hit-and-miss approach of candidate variant studies is employing a gene-wide approach focusing on variations along the *CLOCK* gene.

The high prevalence of depression coupled with the remarkable lack of efficacy of currently available antidepressive medications leaving ~30–35% of patients treatment resistant (25) on the one hand reflects the lack of in-depth understanding of the etiological processes in the background of depression, and on the other hand highlights the need for understanding novel processes and identifying novel molecular targets for intervention. Furthermore, given the well-known heterogeneity of depressive disorders not only on the clinical-symptomatic level, but also in the neurobiological and genetic background of such divergent clinical manifestations, understanding genetic background of various processes, such as circadian disruption, playing a role in the development of different depressive symptoms and syndromes may also help subtypization and, in the end, precision and personalized treatments of depression.

The aim of the present study was to investigate the association between variation in the *CLOCK* gene, lifetime depression, and current depressive symptoms in interaction with childhood adversities and recent negative life events in a general European population.

## Materials and Methods

### Study Sample

The present study was part of the NewMood study funded by the European Union (New Molecules in Mood Disorders, Sixth Framework Program of the EU, LSHM-CT-2004-503474). Seven hundred sixty-seven non-related participants (238 males, 529 females) of self-reported European white ethnic origin aged between 18 and 60 years were recruited from the general population through advertisements, a website and general practices; provided self-reported data on sociodemographic factors including age and gender, as well as lifetime and current depression, and early childhood adversities and recent negative life events occurring in the past year; and provided genetic data by a saliva sampling kit.

NewMood aimed to recruit participants with a diverse exposure to different types on environmental influences and adversities and with diverse socioeconomical background to allow for generalisability of results to real-life settings. Furthermore, as NewMood focused on a general population with a continuum approach to affective symptoms and disorders, our sample included previously depressed, currently depressed and never depressed participants as well. A detailed description of the study population is provided in previously published reports (26–28). The study was carried out in accordance with the Declaration of Helsinki, and it was approved by the Scientific and Research Ethics Committee of the Medical Research Council, Budapest, Hungary. All participants provided written informed consent prior to participating in the study.

### Phenotypes

The present study focused on measuring two aspects of depression and two types of stressors. Lifetime depression (DEP) was ascertained based on self-report using a background questionnaire. This measure to capture the lifetime presence of major depression has been validated previously with face-to-face structured diagnostic interviews (SCID-I) within a subpopulation of our sample, yielding a 91.7% sensitivity and 89.8% specificity (28).

Current level of depression (BSI-Depression) was measured using the Brief Symptom Inventory (29), a questionnaire measuring psychopathological symptoms in several scales with items scored between 0 and 4 depending on the distress caused. For the present study, only the depression subscale score was used to reflect the actual levels of depression, calculated as the sum of depression and additional item scores divided by the number of completed items. Use of BSI-Depression to capture actual depressive symptom severity has been validated in a previous study in a subsample of our population using the Montgomery-Asberg Depression Rating Scale (MADRS) administered by trained interviewers (28).

The two types of stressors measured in our study included early childhood adversities (CHA) as distal and etiological stressors and recent negative life events (RLE) occurring in the past year as proximal, trigger stressors. The early childhood adversity measure was derived from the Childhood Trauma Questionnaire (CTQ) (30), and included four items referring to emotional and physical abuse and emotional and physical neglect, and two items about the loss of parents. This short childhood adversity measure has previously been validated with the 28-item CTQ within a subpopulation of our sample, yielding a high correlation between the original and derived measures (28). The sum of item scores was used in the analyses. Recent stressful life events (RLE) occurring in the past year related to financial difficulties, illnesses/injuries, personal problems, and intimate relationship or social network difficulties were measured using the List of Threatening Experiences (31, 32). The number of recent negative life events (RLEs) was used in the statistical analyses.

### Genotyping and Imputation

Participants provided buccal mucosa cells collected by a cytology brush (Cytobrush plus C0012, Durbin PLC). Genomic DNA was extracted according to the protocol of Freeman et al. (33). Genotyping was performed by Illumina's CoreExom PsychChip. All laboratory work was performed under the ISO 9001:2000 quality management requirements and was blinded with regard to phenotype. Variants were positioned on the genome based on GRCh37/hg19. SHAPEIT was used to determine haplotype information, then missing genotypes were imputed using IMPUTE2 on the *CLOCK* gene with boundaries extended by 10 kilobase pairs on both sides. Imputation and subsequent filtering were carried out in line with multiple quality control steps (34), except that missingness rate (MR), Hardy-Weinberg equilibrium (HWE) and minor allele frequency (MAF) steps were limited to SNPs within the *CLOCK* gene. Variants with an imputation score certainty <0.7 or info <0.5 were excluded.

### Statistical Analyses

Plink v1.90 was used to calculate MR (<0.05), HWE (>1 × 10^−5^) and MAF (>0.01) as part of quality control steps prior to the analyses; for clumping; and for building linear and logistic regression models to test for main and interaction effects of genetic variation in the *CLOCK* gene. Analyses were supported by scripts individually written in R 3.0.2 (35). R was also used to illustrate the effects of significant findings (version 4.0.3 with the ggplot2 package). Descriptive statistics were run using IBM SPSS Statistics 25.

**Figure 1:**
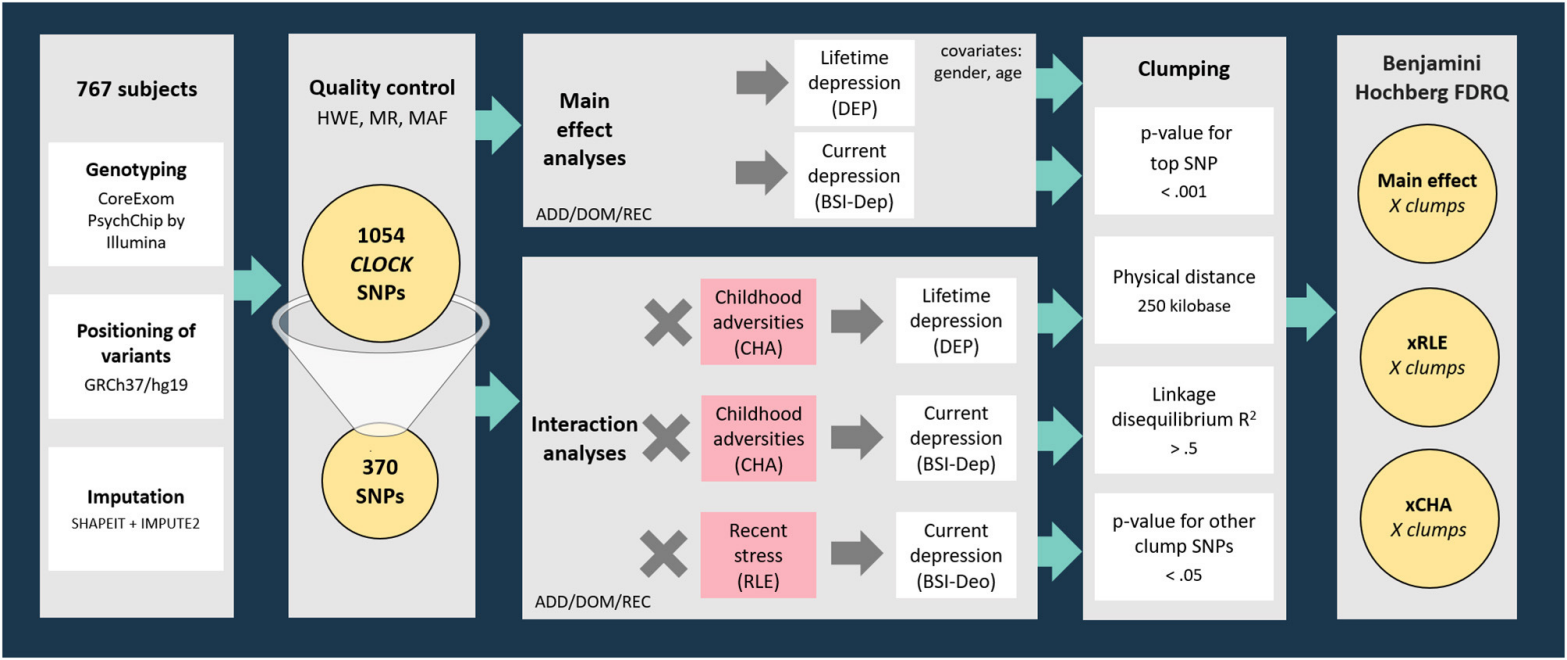
Methods of investigating the effects of variation in *CLOCK* in interaction with childhood adversities and recent life events on lifetime and current depression: study population, quality control steps, and statistical analyses. RLE, recent life events; CHA, Childhood adversities; BSI-Dep, Brief Symptom Inventory depression score; ADD, additive model; DOM, dominant model; REC, recessive model; HWE, Hardy-Weinberg Equilibrium; MR, missingness rate; MAF, minor allele frequency; DEP, lifetime depression; FDRQ, False Discovery Rate Q value.

Genotyping provided a dataset incorporating 1054 SNPs in the region of *CLOCK* gene (with boundaries extended by 10 kb) available in the NewMood database. Three hundred and seventy SNPs survived quality control steps were analyzed with linear (for current depression with BSI-dep as the outcome variable) and logistic (for lifetime depression with DEP as the outcome variable) regression models to test for main effects of *CLOCK* variation on lifetime and current depression. To test for gene-environment correlation (rGE) effects, the main effects of *CLOCK* variants surviving quality control on childhood adversities (CHA) and recent life events (RLE) were also analyzed in linear regression models. After tests for main effects of genetic variants on the outcome variables, gene × environment interaction models with early childhood adversities (CHA) and recent negative life events (RLE) were also run. Regression data on all *CLOCK* SNPs surviving the quality control in all models are shown in Supplementary Tables 1–4). Regression models were in the next step followed by a clumping procedure both for main effect and for GxE interaction effects based on linkage disequilibrium (LD) estimates between the SNPs using the CLUMP function in Plink. The four parameters used for clumping included: (1) maximum *p*-value of the clump's top SNP was set at 0.001; (2) physical distance with top SNP was 250 kilobase; (3) minimum linkage disequilibrium *R*^2^ with top SNP was 0.5; and (4) maximum *p*-value for the clump's other SNPs was 0.05. Results of top SNPs, that is, the most significant SNP representing correlated SNPs in individual clumps are reported (Figure 1).

All analyses, including main effect and interaction effect models, were run according to additive, dominant, and recessive models. Age and gender were covariates in all Plink regression models. When testing an SNP × CHA/RLE interaction effect, main effects of both the SNP and CHA/RLE were also included as covariates in the model. Nominal significance threshold was *p* < 0.05. To correct for multiple comparisons in analyses for each of the above outcome variables, Benjamini-Hochberg False Discovery Rate (FDR) Q-values were calculated; results with a *Q* ≤ 0.05 were considered significant.

The data presented in this study are openly available in FigShare at https://doi.org/10.6084/m9.figshare.14258567.v1 (36).

## Results

### Descriptive Statistics

Descriptive statistics of our study sample are provided in Table 1.

**Table 1:** Descriptive statistics of the study sample.

Gender	n	%		
Male	238	21.76%		
Female	529	48.36%		
	*Minimum*	*Maximum*	*Mean*	*SEM*
Age	18	60	30.724	0.375
Clinical data	*n*	*%*		
Subjects reporting lifetime depression	166	21.64%		
Subjects reporting previous suicide attempts or self-harm	35	4.56%		
	*Minimum*	*Maximum*	*Mean*	*SEM*
BSI-depression score	0	4	0.546	0.024
Environmental influences	*Minimum*	*Maximum*	*Mean*	*SEM*
Childhood adversity	0	15	2.761	0.104
Recent negative life events	0	8	1.091	0.042
Sociodemographic descriptors	*n*	*%*		
**Employment status**				
Working full-time Working part-time Student Retired Housewife/househusband Unemployed	405 27 295 13 12 15	52.80% 3.52% 38.46% 1.69% 1.56% 1.95%		
**Marital status**				
Single Married Cohabiting Divorced Separated Widowed Data missing	360 251 98 35 11 7 5	46.94% 32.72% 12.78% 4.56% 1.43% 0.91% 0.65%		
**Financial situation**				
Living comfortably Just getting by Not able to get along Data missing	495 259 5 8	64.54% 33.76% 0.65% 0.91%		

*BSI-depression score, current depressive symptom scores as measured by Brief Symptom Inventory; SEM, standard error of the mean*.

### Main Effects of Variation in *CLOCK* on Current Depressive Symptoms and Lifetime Depression

Linear and logistic regression models on BSI-Dep (for current depression) and DEP (for lifetime depression), respectively, identified a few SNPs with a nominally significant main effect, but significant clumps could not be formed, furthermore, all *p*-values exceeded the maximum threshold for top SNP (*p* = 0.001) [Supplementary Tables 1, 2 for lifetime depression (DEP) and current depressive symptoms (BSI-Dep, respectively), but significant clumps for main genetic effects could not be identified]. Nevertheless, top SNPs of significant clumps emerging in the interaction models were tested in main effect models for lifetime and current depression as well, the results of which are shown in Table 2.

**Table 2:** Main effects of *CLOCK* variants emerging as top SNPs in the interaction models on lifetime depression and on current depression (BSI-dep) severity.

		**Additive**					**Dominant**					**Recessive**				
	**Model**	**β**	**95% C.I**.		***p*-value**	**FDRQ**	**β**	**95% C.I**.		***p*-value**	**FDRQ**	**β**	**95% C.I**.		***p*-value**	**FDRQ**
rs6828454	Lifetime depression	1.122	0.883	1.426	0.3482	0.497	1.034	0.710	1.506	0.8605	0.906	1.342	0.898	2.005	0.1514	0.293
	BSI depression	0.015	−0.050	0.079	0.6571	0.773	0.031	−0.07	0.133	0.5456	0.668	0.006	−0.106	0.118	0.9189	0.951
rs711533	Lifetime depression	1.524	1.114	2.084	*0.0084*	**0.042**	1.497	1.045	2.144	*0.0279*	0.084	2.945	1.152	7.530	*0.0241*	0.080
	BSI depression	0.070	−0.019	0.159	0.1233	0.264	0.074	−0.024	0.174	0.1463	0.293	0.129	−0.171	0.431	0.3988	0.532
rs6825994	Lifetime depression	1.465	1.149	1.868	*0.0021*	**0.015**	1.648	1.145	2.371	*0.0071*	**0.039**	1.730	1.102	2.715	*0.0171*	0.064
	BSI depression	0.058	−0.009	0.124	0.0913	0.211	0.086	−0.010	0.181	0.0757	0.182	0.059	−0.074	0.192	0.3822	0.533
rs6850524	Lifetime depression	1.351	1.061	1.721	*0.0149*	0.059	1.535	1.048	2.248	*0.0279*	0.088	1.470	0.963	2.242	0.0741	0.185
	BSI depression	0.063	−0.003	0.129	0.0608	0.159	0.106	0.007	0.205	0.0358	0.102	0.054	−0.067	0.175	0.3832	0.523

*BSI-current depressive symptom scores as measured by Brief Symptom Inventory. DEP-self-reported lifetime depression. ADD, DOM, REC, additive, dominant and recessive heritability models. FDR, false discovery rate; Italic type shows nominally significant p-values, bold type denotes significant Q-values surviving correction for multiple testing (P < 0.05, FDR Q < 0.05)*.

### Gene-Environment Correlation (rGE): Main Effects of Variation in *CLOCK* on Recent Life Events (RLE) and Childhood Adversities (CHA)

Main effect linear regression models on recent life events (RLE) and childhood adversities (CHA) did not identify any nominally significant SNPs (Supplementary Tables 5, 6 for RLE and CHA, respectively) suggesting no gene-environment correlation (rGE) effects in case of either early or recent adversities. Linear regression results for potential rGE effects on RLE or CHA in case of top SNPs of significant clumps emerging in the interaction models are also shown in Table 3.

**Table 3:** Main effects of *CLOCK* variants emerging as top SNPs in the interaction models on recent stressful life events and childhood adversities (rGE models).

		**Additive**				**Dominant**				**Recessive**			
	**Model**	**β**	**95% C.I**.		***p*-value**	**β**	**95% C.I**.		***p*-value**	**β**	**95% C.I**.		***p*-value**
rs6828454	RLE	0.020	−0.094	0.133	0.7341	0.061	−0.117	0.239	0.5019	−0.015	−0.212	0.181	0.8788
	CHA	−0.146	−0.423	0.131	0.3012	−0.269	−0.704	0.166	0.2251	−0.111	−0.592	0.369	0.6504
rs711533	RLE	0.037	−0.119	0.193	0.6441	0.0505	−0.125	0.226	0.5722	−0.037	−0.566	0.492	0.8910
	CHA	−0.224	−0.604	0.157	0.2494	−0.263	−0.691	0.164	0.2279	−0.177	−1.470	1.115	0.7879
rs6825994	RLE	0.005	−0.113	0.123	0.9337	0.073	−0.095	0.241	0.3967	−0.122	0.120	−0.356	0.113
	CHA	0.011	−0.276	0.298	0.9417	0.113	−0.295	0.522	0.5874	−0.122	−0.356	0.113	0.3085
rs6850524	RLE	0.046	−0.071	0.164	0.4404	0.110	−0.067	0.287	0.223	−0.008	0.110	−0.223	0.207
	CHA	−0.014	−0.302	0.275	0.9245	0.039	−0.395	0.473	0.8597	−0.008	−0.223	0.207	0.9439

*RLE, recent life stress; CHA, Childhood Adversity. ADD, DOM, REC, additive, dominant, and recessive heritability models*.

### Gene x Environment Effects of Variation in *CLOCK* on Current Depressive Symptoms: Interaction With Recent Life Events (RLE)

Our analyses for interaction with recent stressful life events (RLE) on current depressive symptoms (BSI-Dep) yielded one significant clump containing 94 SNPs (Supplementary Table 3) and with top SNPs rs6825994 for dominant and rs6850524 for additive models, both surviving correction for multiple testing (Table 4).

**Table 4:** Interactions of *CLOCK* top SNPs rs6828454, rs711533, rs6850524, and rs6825994 with recent negative life events (RLE) and childhood adversity (CHA) on current depression (BSI-Dep) and lifetime depression (DEP) (GxE models).

		**Additive**					**Dominant**					**Recessive**				
	**Model**	**β**	**95% C.I**.		***p***	**FDRQ**	**β**	**95% C.I**.		***p*-value**	**FDRQ**	**β**	**95% C.I**.		***p***	**FDRQ**
rs6828454	xRLE on BSI	0.017	−0.036	0.071	0.5240	0.655	0.046	−0.044	0.136	0.3172	0.476	0.003	−0.083	0.089	0.9469	0.947
	xCHA on BSI	0.034	0.013	0.055	*0.0015*	**0.0145**	0.032	−0.000	0.065	0.0510	0.1390	0.066	0.028	0.104	*0.0006*	**0.0118**
	xCHA on DEP	0.992	0.920	1.070	0.8424	0.903	0.974	0.865	1.096	0.6606	0.762	1.016	0.887	1.164	0.8143	0.905
rs711533	xRLE on BSI	0.088	0.018	0.158	0.0141	0.060	0.095	0.016	0.174	0.0188	0.066	0.139	−0.085	0.364	0.2244	0.396
	xCHA on BSI	0.053	0.022	0.084	*0.0008*	**0.0092**	0.058	0.024	0.092	*0.0008*	**0.0114**	0.066	−0.054	0.186	0.2815	0.4692
	xCHA on DEP	1.045	0.934	1.168	0.4458	0.581	1.070	0.944	1.213	0.2878	0.467	0.892	0.605	1.315	0.5621	0.675
rs6850524	xRLE on BSI	0.098	0.044	0.152	*0.0004*	**0.013**	0.127	0.044	0.210	*0.0028*	**0.019**	0.132	0.037	0.228	*0.0066*	**0.040**
	xCHA on BSI	0.014	−0.008	0.036	0.2160	0.3927	0.017	−0.016	0.050	0.3099	0.4768	0.020	−0.020	0.061	0.3192	0.4671
	xCHA on DEP	1.057	0.973	1.148	0.1866	0.350	0.986	0.872	1.114	0.8173	0.892	1.230	1.047	1.445	0.0117	*0.054*
rs6825994	xRLE on BSI	0.091	0.034	0.147	*0.0017*	**0.015**	0.141	0.064	0.219	*0.0004*	**0.023**	0.061	−0.055	0.176	0.3046	0.481
	xCHA on BSI	0.013	−0.010	0.036	0.2622	0.4495	0.024	−0.008	0.056	0.1408	0.2913	0.002	−0.043	0.047	0.9237	0.9394
	xCHA on DEP	1.028	0.946	1.117	0.5139	0.656	0.982	0.873	1.104	0.7614	0.862	1.145	0.967	1.357	0.1171	0.260

*BSI–current depressive symptom scores as measured by Brief Symptom Inventory. DEP–self–reported lifetime depression. ADD, DOM, REC, additive, dominant and recessive heritability models; CHA, Childhood Adversity; RLE, recent life stress; FDR, false discovery rate. Italic type shows nominally significant p-values, bold type denotes significant Q-values surviving correction for multiple testing (P < 0.05, FDR Q < 0.05). In case of all top SNPs statistical parameters are shown for all analyses, not only for those models where they emerged as top SNPs, thus gray shading indicates results for the models where the given SNP was top SNP*.

**Figure 2:**
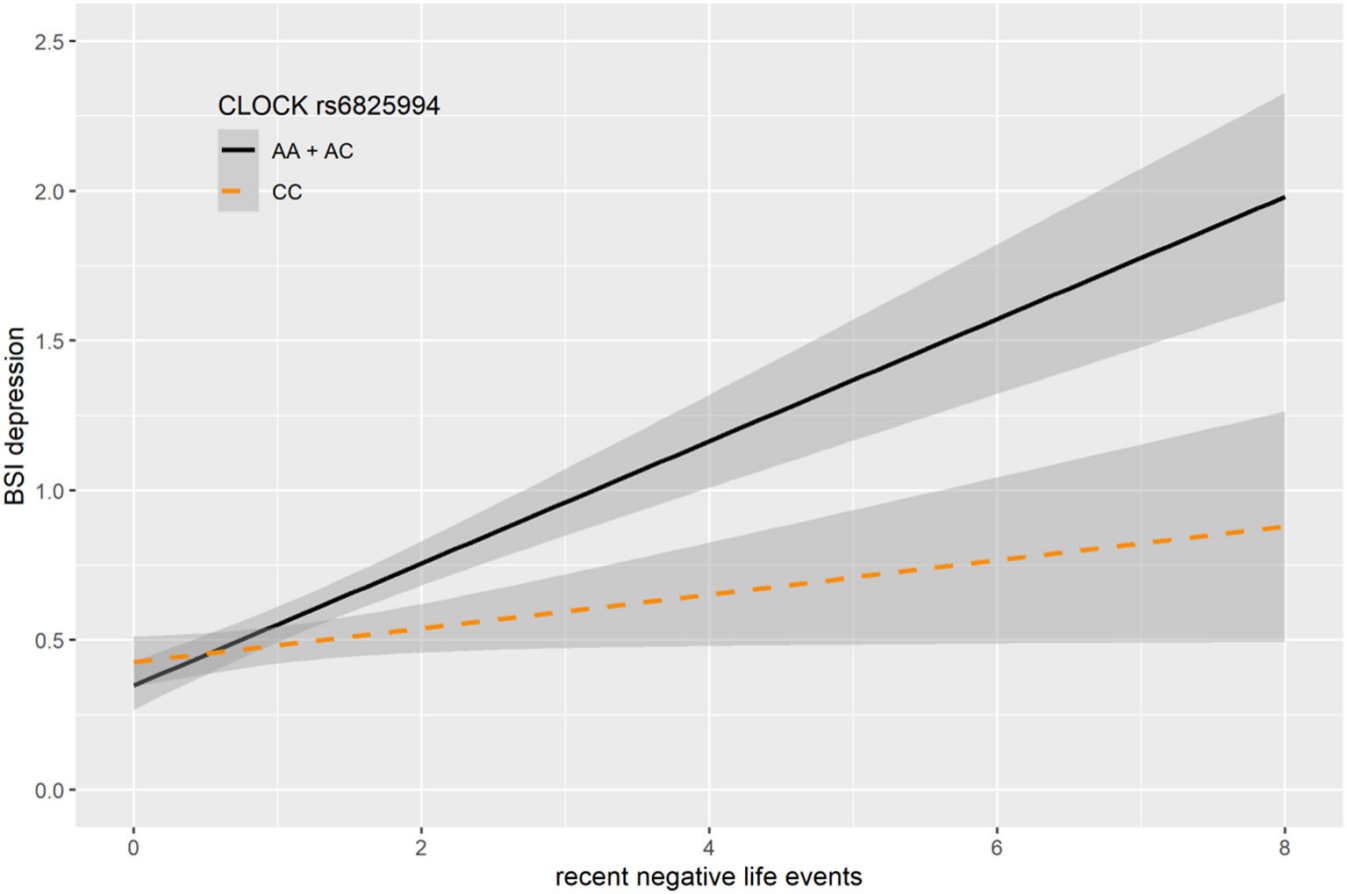
Linear regression indicated a significant interaction between top SNPs rs6825994 of a clump containing 94 SNPs in the *CLOCK* gene and exposure to recent life events (RLE) on current depressive symptoms (BSI-Dep) in the dominant model, with the minor A allele as a risk allele. Linear regression indicated a significant interaction between *CLOCK* rs6825994 genotype and recent stressful life events (RLE) on current depression scores according to the dominant (*p* = 0.0004, FDR *Q* = 0.023, as top SNP) and additive (*p* = 0.0017, FDR *Q* = 0.015) models. Presence of the minor A allele was associated with higher depression scores in subjects exposed to more severe recent life events conveying a risk effect. On the vertical axis weighted depression (BSI-Dep) scores are shown. The horizontal axis shows recent life events (RLE) occurring within the past year as measured by the List of Threatening Experiences (31). BSI-Brief symptom inventory. Gray shading denotes 95%CI.

In case of rs6825994, we found a nominally significant interaction effect with recent negative life events (RLE) on BSI-depression in dominant (*p* = 0.0004) model as the lead SNP, and its effect in the additive model was also nominally significant (*p* = 0.0017), both of which remained significant after correction for multiple testing (FDRQ_add_ = 0.0149 and FDRQ_dom_ = 0.0232, respectively) (Table 4). Subjects carrying minor A allele of rs6825994 had significantly higher current depression scores when exposed to recent stressful life events suggesting the minor allele to be a risk allele (Figure 2).

**Figure 3:**
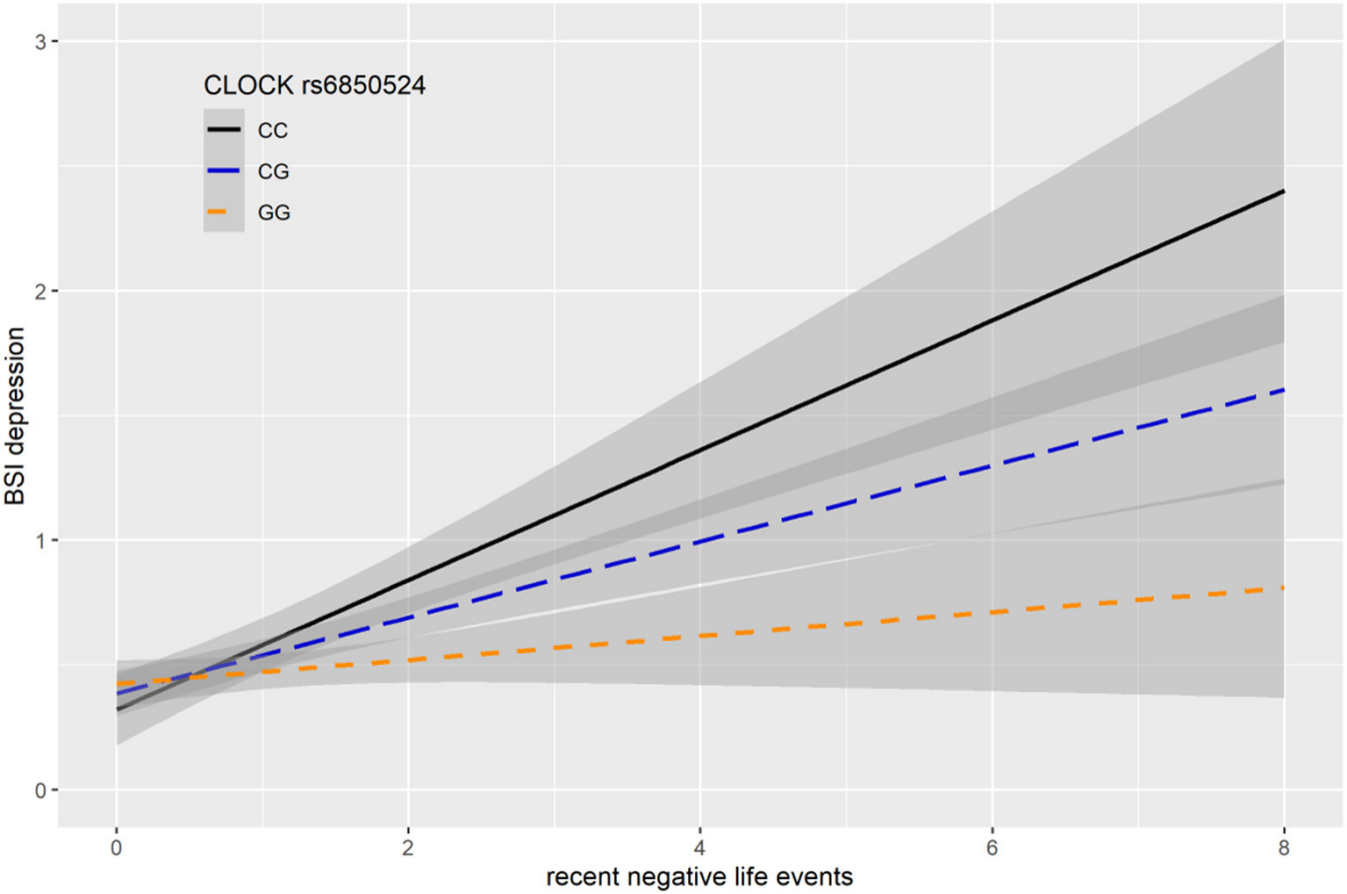
Linear regression indicated a significant interaction between top SNP rs6850524 of a clump containing 94 SNPs in the *CLOCK* gene and exposure to recent life events (RLE) on current depression symptoms (BSI-dep) in the additive model, with the minor C allele as a risk allele. Linear regression indicated a significant interaction between *CLOCK* rs6850524 genotype and recent stressful life events (RLE) on current depression scores according to the additive model as top SNP (*p* = 0.0004, FDR *Q* = 0.013). Presence of the minor C allele was associated with higher depression scores in subjects exposed to more severe recent life events conveying a risk effect. On the vertical axis weighted depression (BSI-Dep) scores are shown. The horizontal axis shows recent life events (RLE) occurring within the past year as measured by the List of Threatening Experiences (31). BSI-Brief symptom inventory. Gray shading denotes 95%CI.

In case of rs6850524 we found nominally significant interaction effects with recent life events (RLE) on BSI-depression as top SNP in the additive model (*p* = 0.0004) which survived correction for multiple testing (FDRQ_add_ = 0.0127). This SNP, although not as a top SNP, was also nominally significant in interaction with recent life events on BSI-depression in dominant (*p* = 0.0028) and recessive (*p* = 0.0066) models which all remained significant after correction for multiple testing (FDRQ_dom_ = 0.0187, FDRQ_rec_ = 0.0398) (Table 4). In these models, presence of the minor C allele was associated with a lower BSI-depression score if the subject was exposed to recent negative life events indicating a risk effect (Figure 3).

### Gene × Environment Effects of Variation in *CLOCK* on Current Depressive Symptoms and Lifetime Depression: Interaction With Childhood Adversities (CHA)

In case of gene x environment interaction models with childhood adversity (CHA) on current depression (BSI-Dep), we identified two clumps with top SNPs rs6828454 and rs711533 (Table 4), both of them containing 9 SNPs (Supplementary Table 4).

**Figure 4:**
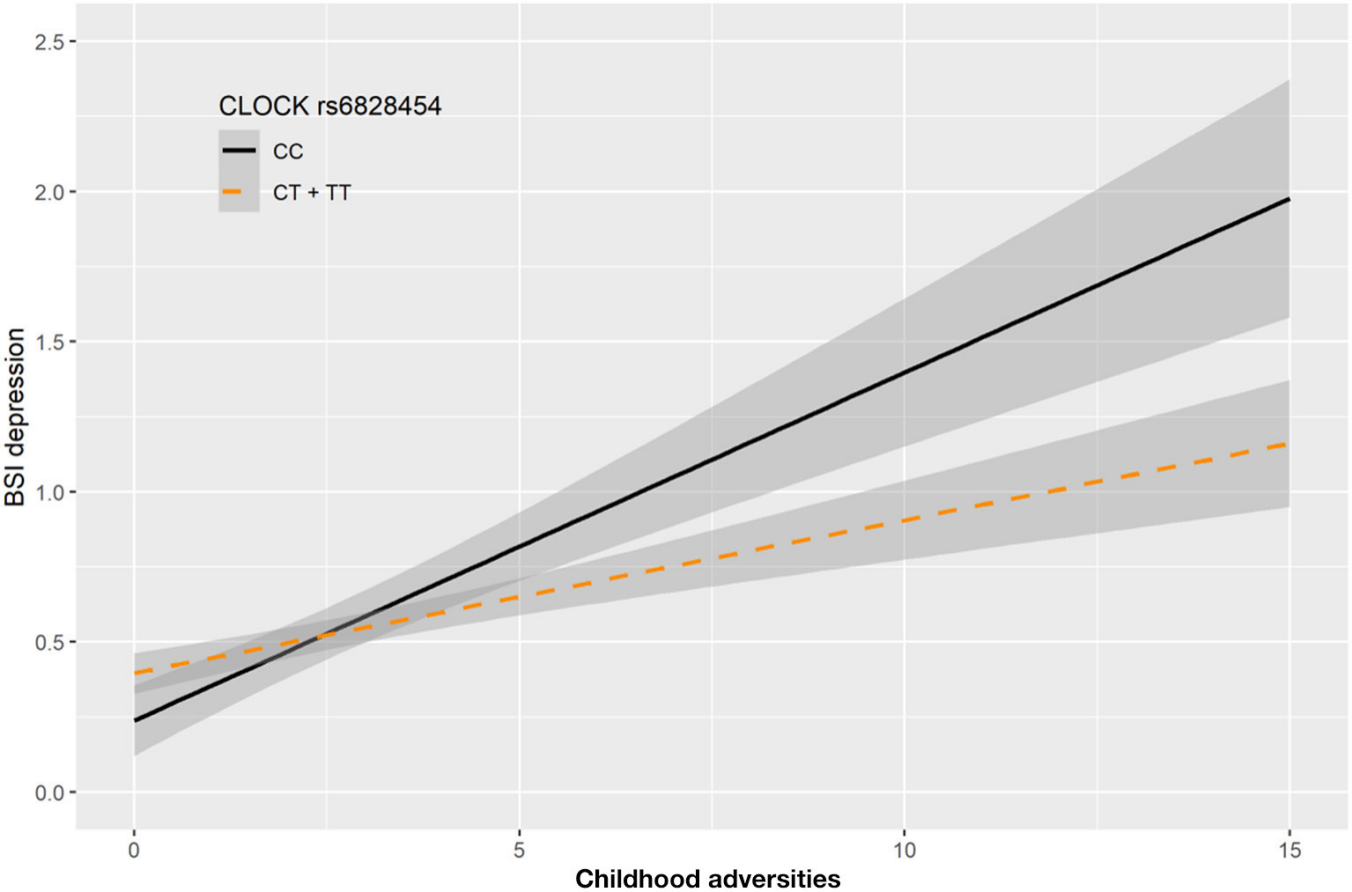
Linear regression indicated a significant interaction between top SNP rs6828454 of a clump containing 9 SNPs in the *CLOCK* gene and exposure to childhood adversities on current depression symptoms (BSI-dep) in the recessive model, with the minor C allele being a risk allele. Linear regression indicated a significant interaction between *CLOCK* rs6828454 genotype and childhood adverse life events (CHA) on current depression scores according to the recessive model as top SNP (*p* = 0.0006, FDR *Q* = 0.0118). Homozygous presence of the minor C allele was associated with higher depression scores in subjects exposed to more severe childhood adverse events conveying a risk effect. On the vertical axis weighted depression (BSI-Dep) scores are shown. The horizontal axis shows childhood adverse life events (CHA) as measured by an instrument derived from the CTQ (30). BSI-Brief symptom inventory. Gray shading denotes 95%CI.

In case of rs6828454 we found a significant interaction effect with childhood adversities (CHA) on current depression (BSI-Dep) in the recessive model as top SNP (*p* = 0.0006, FDRQ = 0.0118). Minor C allele carriers reported significantly higher current depression levels when exposed to moderate or severe childhood adversity indicating the minor allele to be a risk allele (Figure 4).

**Figure 5:**
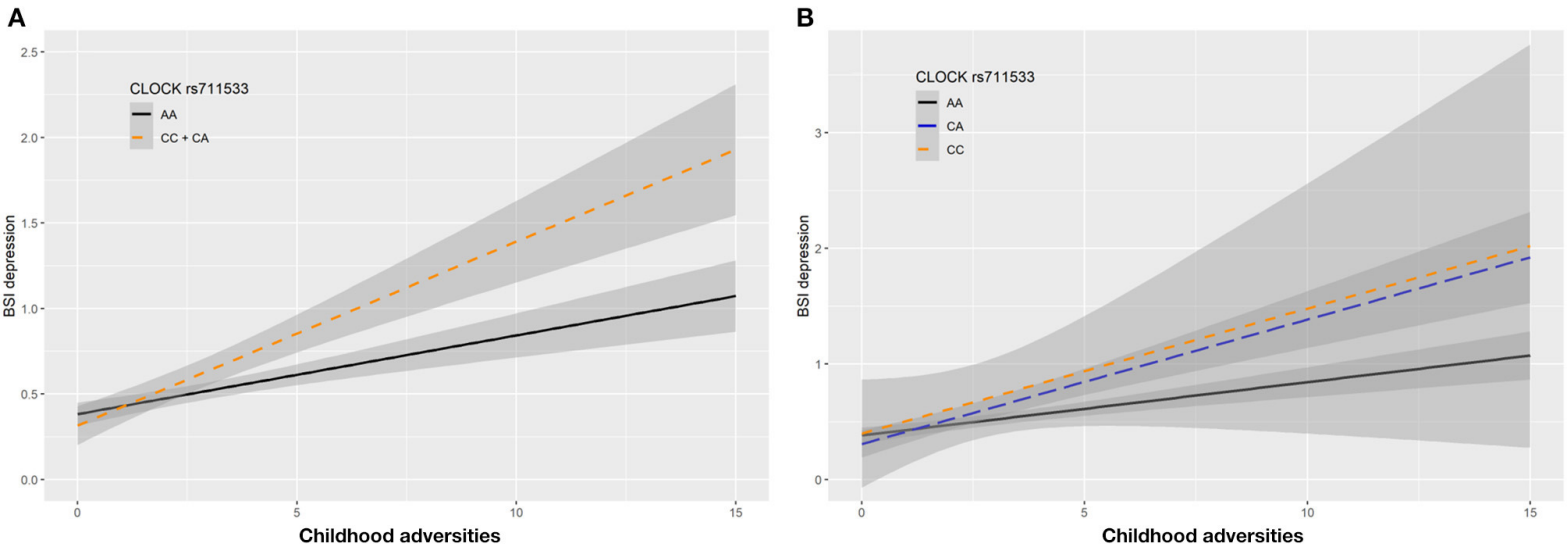
Linear regression indicated a significant interaction between top SNP rs711533 of a clump containing 9 SNPs in the *CLOCK* gene and exposure to childhood adversity (CHA) on current depression symptoms (BSI-Dep) in the dominant **(A)** and additive **(B)** models, with the minor C allele being a risk allele. Linear regression indicated a significant interaction between *CLOCK* rs711533 genotype and childhood adverse life events (CHA) on current depression scores according to the dominant and additive models as top SNP (*p* = 0.0006, FDR *Q* = 0.0118). Presence of the minor C allele was associated with higher depression scores in subjects exposed to more severe childhood adverse events conveying a risk effect. On the vertical axis weighted depression (BSI-Dep) scores are shown. The horizontal axis shows childhood adverse life events (CHA) as measured by an instrument derived from the CTQ (30). BSI-Brief symptom inventory. Gray shading denotes 95%CI.

In case of rs711533 we found significant interaction effects with CHA on current depression (BSI-Dep) as top SNP in both additive (*p* = 0.0008, FDRQ = 0.0092) and dominant (*p* = 0.0008, FDRQ = 0.0114) models remaining significant after correcting for multiple testing (Table 4). Subjects carrying the minor C allele scored significantly higher on BSI-depression scale when exposed to childhood maltreatment reflecting a risk effect for the minor allele (Figure 5).

Interaction with childhood adversity did not have a significant effect on lifetime depression in case of any SNPs, therefore significant clumps could not be calculated (Table 4).

### *In silico* Characterization and Functional Prediction of Identified Top SNPs rs6828454, rs711533, rs6825994, and rs6850524 as Well as SNPs in the Clumps Showing a Significant Effect on Depression

**Figure 6:**
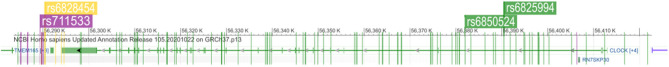
Genomic location of significant SNPs identified in the clumping procedure, in the analysis of variation in *CLOCK* in interaction with recent life events and early childhood adversities on current depressive symptoms. In interaction with recent life events (RLE), our analyses identified one clump containing 94 SNPs with top SNPs rs6825994 and rs6850524 (for dominant and additive models, respectively) on current depressive symptoms (BSI-Dep), marked in green. In interaction with childhood adversities (CHA), two clumps, each with 9 SNPs and top SNPs rs6828454 and rs711533 were identified on current depression symptoms which are shown in yellow and purple, respectively. UCSC Genome Browser on Human Feb. 2009 (GRCh37/hg19) was used to visualize the location of polymorphisms on *CLOCK* gene.

Genomic location of significant SNPs and top SNPs identified in the clumping procedure are shown in Figure 6. To detect the functional effect of the top significant SNPs, we utilized FuncPred tool (https://snpinfo.niehs.nih.gov). Two SNPs (rs28448438, rs28463765) are located in the transcriptional-factor-binding site of *CLOCK* gene, furthermore, rs726967 is located in the microRNA-binding site. Among the significant polymorphisms, several SNP showed high regulatory potential and conservation score.

In the literature exploratory analyzes on SNPedia (https://www.snpedia.com/), ClinVar (https://figshare.com/articles/dataset/CLOCK_stress_depression/14258567), GWAS Catalog (https://www.ebi.ac.uk/gwas/), we found some relevant sources related to the following SNPs: rs6853192, rs11726609, rs11133399, rs4865010, rs1000254, rs2412646, rs3736544, rs6811520, rs11931061, rs3817444, rs6832769, rs726967, and rs11735267. Results are shown in Supplementary Table 7 and discussed in the Discussion part.

## Discussion

In our study investigating the effect of variation in the *CLOCK* gene with a linkage disequilibrium-based clumping method, we found no clumps of SNPs with a significant main effect either on lifetime depression or current depressive symptoms. However, we have identified significant clumps of SNPs in interaction with both early childhood adversities and recent negative life events on current depressive symptoms, but not in case of lifetime depression. While our results confirm the role of *CLOCK* variation in emergence of depressive symptoms, they also indicate that this effect is observable only in case of exposure to stress. Notably, we found that clumps of SNPs in the *CLOCK* gene interacted with both distal childhood traumas and adversities, which contribute to the emergence of a diathesis promoting susceptibility for affective disorders, and recent, proximal stressors, which have a role in triggering onset of the actual symptoms or illness episodes based on a diatheses. Finally, we detected no gene-environment correlation effects suggesting that *CLOCK* variation does not influence risk of exposure to early or recent adversities.

### The Involvement of Circadian Disruption in Depression

Circadian rhythms regulate a multitude of physiological processes coordinating them both with each other and with the external environment, integrating outer sensory information, and environmental cues with internal physiological and psychological states, thus also influencing human cognition, affect, and behavior, which suggests that dysregulated or disturbed circadian rhythms are involved in the etiopathology of mental and mood disorders as well (6, 19). The presence of circadian abnormalities in depression have been evidenced for a long time with alterations in depressed patients in the circadian rhythmicity of somatic functions including body temperature, blood pressure, or urine metabolite excretion; altered hormone rhythms including prolactin, cortisol, GH, thyrotropin, and melatonin; and in the diurnal fluctuation of symptoms of depression including alteration in sleep-wake cycles, timing and structure of sleep, appetite, or social rhythms (8–14, 37). While in clinical studies risk and severity of depression correlated with the degree of circadian rhythm misalignment (19, 38), there is no definitive evidence whether circadian dysregulation precedes and plays a causative role in, or follows and results from, or merely coincides with depression, however, the complex relationship between the circadian system and mood disorders appears to be bidirectional (7, 39). Nevertheless, conditions leading to circadian rhythm disruption such as shift work may precipitate mood symptoms in those susceptible (40, 41), and in post-mortem brain studies in depressed patients severe disruption and desynchronization of daily rhythmic gene expression patterns were reported (42). One possible linking factor between the circadian system and mood disorders is that neural systems playing a key role in affective illness including the HPA-axis, limbic regions, and monoamine neurotransmitter household are under circadian regulation, and alterations in the biological clock could lead to neurobiological changes in neurotransmitter systems triggering depressive states (12, 14, 42–44). While variation in clock genes encoding elements of circadian rhythm mechanisms is associated with smaller alterations of circadian behaviors in healthy subjects, it appears to have a more pronounced impact on psychopathological features in mood disorder patients affecting key clinical and course features such as timing of disease onset and recurrence and treatment response (45–47).

### Variation in the *CLOCK* Gene Does Not Directly Impact Lifetime Depression or Current Depressive Symptoms

Our findings indicate that variation in the *CLOCK* gene exerts no significant main effect either on lifetime depression, or current depressive symptoms. The *CLOCK* gene, located at chr4q12, is one of the chief genes in the endogenous master clock system playing a key role in the formation of circadian rhythms. Genomic variation in *CLOCK* gene in common polymorphisms with a MAF of ≥1% in 1000 Genomes Project (48) comprises 406 SNPs for African, 271 for European, 278 for Asian, and 277 for American continental populations (49). The *CLOCK* gene, as part of CLOCK(NPAS2)/ARNTL complex regulates rhythmic transcription of clock-controlled genes (CCG) in several tissues, including at least 15% of mammalian transcripts many of which gene expression patterns are disrupted in MDD (11, 19, 50, 51). The *CLOCK* machinery could provide mechanisms for control of circadian gene expression and responsivity to stimuli on cellular levels influencing activity of brain structures which control emotions and behavior (47). Furthermore, the function of *CLOCK* protein as a transcription factor and histone acetyltransferase also implies that genetic and epigenetic variations could contribute to physiological changes possibly leading to altered susceptibility of psychiatric disorders including depression (49). *CLOCK* in the mammalian brain is expressed in several brain structures beyond the master clock, the nucleus suprachiasmaticus, including the cortex (52), and molecular and behavioral studies support that *CLOCK* gene plays an important role in neuronal function involved in the regulation of several pathways implicated in psychiatric disorders and its genetic manipulation leads to marked changes in neurotransmitter activity and behavior. For example, *CLOCK* regulates expression of neurogenic transcription factors influencing the differentiation of adult neural stem cells in mice (53), controls transcription of tyrosine hydroxylase and cholecystokinin and other regulators of monoaminerg transmission (54), and disruption of *CLOCK* gene function leads to alterations in glutamatergic and GABAergic signaling (49, 55). Animal experiments on *CLOCK* gene in psychiatric condition-related behaviors have shown biological plausibility and promising findings (3), while in humans *CLOCK* variations have been implicated in susceptibility to phenotypes of common psychiatric disorders including autism spectrum disorders, schizophrenia, attention deficit/hyperactivity disorder, substance use disorder, major depressive disorder, bipolar disorder, and anxiety (49).

Several studies suggested an association between *CLOCK* and mood disorders, however, results are inconsistent in case of unipolar depression (56, 57). Previous studies employing a candidate variant approach focused only on a few variants and mainly on rs1801260, speculated to affect mRNA, which has been found to be associated with evening preference and a substantial, 10–44-min delay in preferred timing for activity and sleep in healthy C carriers (58). In some studies rs1801260 has been shown to be associated with clinical features mostly in case of bipolar disorder, especially with sleep problems including increased occurrence of lifetime and episode-related insomnia (59), higher recurrence of initial, middle and late insomnia in both MDD and BD patients (60), number and recurrence of manic episodes (17), and appetite disturbances in women (49), but a meta-analysis could not confirm an association between rs1801260 and either unipolar or bipolar mood disorder (61). These findings suggest that this variant is not associated with mood symptoms itself but only with certain, mainly sleep-related symptoms accompanying mood disorders. Other, less numerous studies focused on other *CLOCK* variants, with a nominally significant relationship between rs11932595 and SSRI efficacy, rs534654 and weight loss and rs12504300 and rs3825148 and SSRI side effects including nausea, constipation and vomiting in a Han Chinese population (11).

In light of the above contradictory findings and the general lack of robust and replicable positive associations between candidate *CLOCK* SNPs and depression, our present findings, investigating variation along the *CLOCK* gene corroborate previous reports suggesting that *CLOCK* does not have a significant direct main effect on lifetime depression or current depressive symptoms (61, 62).

### *CLOCK* Variation Mediates the Effect of Childhood Adversities and Recent Negative Life Events on Current Depressive Symptoms

More importantly, in spite of a lack of a main effect of *CLOCK* variation on depressive phenotypes, we did detect a robust effect of several clumps of SNPs on current depressive symptoms in interaction with both distal, early childhood, and proximal, recent stressors, which is in line with our paradigm postulating that the majority of genes impact depression by increasing susceptibility toward the negative effects of stress rather than exerting a direct effect (16, 21).

According to the social Zeitgeber theory, mood disorders are triggered by life stressors disrupting normal routines and circadian rhythms thus altering mood and biological rhythms (63). Several depression-relevant brain functions are controlled simultaneously by the circadian clock and stress response systems which are themselves closely connected (7). The relationship between stress and the circadian rhythm regulation is bidirectional, with both circadian rhythms impacting stress response and the effects of stress impacting regulation of circadian rhythms. Diurnal cycles of glucocorticoids, regulating various stress response-related physiological and behavioral responses, are one of the most prominent endocrine manifestations of circadian rhythms and themselves play a role in synchronizing peripheral and circadian oscillators with promoters of several clock genes containing glucocorticoid responsive elements (GREs) (7, 64, 65). Furthermore, many stress-inducible genes are also under control of the circadian clock (7). The circadian clock and stress response systems show an extensive overlap and regulate multiple systems which control cognition, affect, reward processing, and other systems and functions implicated in depression (7), suggesting that modulation of glucocorticoid-mediated stress response may constitute a common mechanism by which circadian clock affects mood disorders (19). Animal studies have shown that chronic mild stress led to anhedonic behavior associated with disturbed diurnal oscillation in circadian gene expression and higher *Clock* expression in the basolateral amygdala (66) and reduced *CLOCK* protein levels in the prefrontal cortex (67). As variants of *CLOCK* gene show differential transcriptional response to glucocorticoids (19), it is possible that variation in the *CLOCK* gene may mediate the effects of stress on the development of depressive symptoms.

Although only a few human studies took stressors into consideration when investigating the effects of *CLOCK* variants, some both in healthy and depressed subjects reported that, similar to our findings, the impact of the investigated variants was detectable only in those exposed to some type of stress. In healthy subjects, presence of the C allele of rs1801260 has been associated with a greater disruption of sleep patterns following stressful life events, and a few studies in mood disorder patients found that this variant was associated with sleep change only in case of prior stressful experiences (10) concluding that environmental stress may increase vulnerability to circadian rhythm disruption (10) and suggesting an interaction between *CLOCK* variants in shaping individual risk for deleterious effects of environmental stress including depression (47). In another study in bipolar disorder patients, a significant interaction between *CLOCK* variants and early stress exposure on hopelessness and suicide in BP patients was reported (47). In a non-clinical Chinese population, G allele of rs11932595 was associated with altered sleep duration only in those exposed to high job stress, but showed no effect in case of low stress, while AA carriers showed less daytime dysfunction under low stress and more in high stress conditions compared to G carriers suggesting not only a gene-environment interaction effect, but also the role of the *CLOCK* as both a vulnerability and resilience gene with both positive and negative effects depending on stress (68).

Our present findings showing that *CLOCK* variation increases depressive symptoms only in those exposed to either early childhood adversities or recent stressful life events extend research suggesting that *CLOCK* gene is involved in the development of depressive symptoms by increasing vulnerability toward the disruptive effects of stress. More importantly, we found that both distal, early stressors, with a diathesis-forming etiological role, and recent negative life events, proximal stressors with a triggering role, interacted with *CLOCK* variation on severity of actual depressive symptoms. The exact mechanisms and phenotypes through which *CLOCK* in interaction with stress influences risk or severity of depression needs further study, but it has been suggested that *CLOCK* could hypothetically directly influence neural activity in brain structures playing a key role in generation and control of emotions and affect thus biasing depressive cognition in depression and resilience to detrimental effects of exposure to early stress (10, 69) and the interplay between *CLOCK* variation could influence the effects of stress on the central nervous system determining both vulnerability to or expression of phenotypes related to depression (7).

### *In silico* Characterization and Functional Prediction of Top SNPs and SNPs in the Significant Clumps

In the final step of our analysis we carried out *in silico* characterization and functional prediction for the SNPs in the three clumps significantly impacting current depressive symptoms in interaction with either childhood adversities or current stressors. The search yielded several relevant findings (Supplementary Table 7). Notably, two SNPs, rs28448438 and rs28463765 are located in the transcriptional binding site of the *CLOCK* gene, while a third SNP, rs726967 is located in the microRNA-binding site possibly influencing transcriptional activity.

Of the four top SNPs, rs6850524, which significantly interacted with recent life events on current depressive symptoms has previously been associated with depression-relevant phenotypes including susceptibility to bipolar disorder (70), sleep quality (71), and risk of sleep disorders (72), which indicates its involvement in affective disorders and their symptomatology. This variant has also been found to be associated with somatic conditions including risk of obesity (73, 74), and non-alcoholic fatty liver disease (75) which may be clinically relevant considering the frequent comorbidity of the above illnesses with depression (76–78).

Among SNPs in the clumps significantly interacting with recent life events symptoms, rs6832769 has been reported to be related to emotional prosociality and agreeableness (79, 80), with response and remission with fluvoxamine in MDD but was not found to be associated with affective disorders (81–84), whereas rs2412646 has been associated with depression-comorbid alcohol use disorders and susceptibility to restless leg syndrome in schizophrenic patients (85–87). Several SNPs in the clumps significantly interacting with recent life events including rs11931061, rs3817444, and rs726967 have been reported to be associated with ADHD (88) or sleep problems, including rs11735267 showing a nominal association with late insomnia (70), and rs6853192 with longer sleep duration in general community sample of African Americans (89). In addition, several SNPs in the clumps interacting with recent life events were associated with risk of somatic conditions, including rs10002541 with abdominal obesity and diabetes (74), rs11726609 with BMI in African Americans (89), rs11133399 with gastric cancer overall survival and recurrence free survival and also a primary risk factor contributing to prognosis (90), rs4865010 with essential hypertension and coronary artery disease (91) and in men with hypertonia with testosterone levels corresponding to an androgenic deficient state itself a risk factor for cardiovascular complications (92), and rs6811520 with myocardial infarction (93), multiple sclerosis (94), male infertility (95).

In case of SNPs in clumps significantly interacting with childhood adversities on current depression, rs3749473 was associated with cognitive aging (96), rs6858749 with habitual sleep duration in interaction with protein intake (97, 98), and rs534654 with ADHD (88), disrupted sleep-wake cycles in BD (70), and development of depression (11).

### Possible Clinical Implications of *CLOCK* Variation in Interaction With Early and Recent Stress in Depression

The association of stress and circadian vulnerability especially in case of mood disorders has significant clinical relevance in case of stress chronotherapy. As circadian disruptions and frequent stress exposure—especially in interaction—amplify the risks of development of a wide range of health-related disorders including mood disorders, simultaneous reduction of circadian and classical stressors including strategies stabilizing endogenous rhythms to counteract circadian perturbations would be beneficial. While the majority of such practices are hardly feasible in the modern lifestyle, there are several effective therapies for depression which act via modulating circadian parameters, including bright light, sleep deprivation, and phase resetting paradigms (12, 19, 99, 100) which improve symptoms within hours (101, 102). Scheduled meals or activity, or specific interventions such as social rhythm therapy to enhance circadian alignment of peripheral and central clocks also offer means of reducing circadian stress to prevent or improve mood disorder symptoms (103). Our study identifying an interaction between *CLOCK* variation and both childhood and recent stressors in the development of depressive symptoms could in the future help predict those at a higher risk for development of depression in case of circadian disruption, and also those who would benefit from chronotherapies for depression.

Furthermore, antidepressants including SSRIs, SNRIs and agomelatine also directly impact circadian rhythms as part of their effects improving depressive symptoms (6, 104–106). For example, fluoxetine normalizes disrupted light-induced entrainment, fragmented ultradian rhythms and altered hippocampal *CLOCK* expression in animal models of depression (107), while agomelatine, a melatonergic antidepressant is hypothesized to resynchronize disrupted circadian rhythms (108, 109). It has also been suggested that *CLOCK* expression may predict efficacy of antidepressants exerting their effects via influencing circadian mechanisms (68). Thus, understanding the role of *CLOCK* variation in depression may help identify new targets for pharmacological treatment possibly via modulating the impact of stress on brain function (7) as well as prediction of efficacy especially in subtypes of depression aiding precision therapy.

### Limitations

When interpreting the impact of our findings, several limitations of our study must also be taken into account. First, both early childhood adversities and recent life events occurring in the past year were assessed retrospectively, and based on self-report of the subjects but not ascertained by other informants, and are thus subject to recall and reporting biases. Second, both lifetime depression and current depression severity was similarly based on a self-reported measure. Third, our population sample was relatively small, consisting approximately two thirds of female subjects, and limited to European white participants. Fourth, scoring childhood adversity and counting the number of recent negative life events does not take into consideration the differing severity and subjective impact of individual life events. Nevertheless, our study also has several strengths, including considering several hundred variants along the *CLOCK* gene with a clumping method rather than individual, hypothesis-based candidate SNPs, employing a dimensional approach capture current depression symptom severity, and using a GxE paradigm with two etiologically different types of stressors.

## Conclusion

In conclusion, the results of our study investigating variation along the *CLOCK* gene with a linkage disequilibrium-based clumping of SNPs support the role of *CLOCK* in the background of depressive symptoms but only in association with recent stress and early adversities, underlining not only the depressogenic importance of circadian disruption but also that it acts in interaction with both early, etiological, and recent, trigger-like stressors. Thus, our findings strengthen the rationale of looking at the circadian system in search of new molecular targets for pharmacological interventions, and by specifically reporting that effects in clock variation are only observable in interaction with stress also helps to explain previous lack of consistent findings.

## Data Availability Statement

The datasets presented in this study can be found in online repositories. The names of the repository/repositories and accession number(s) can be found at: https://figshare.com/articles/dataset/CLOCK_stress_depression/14258567.

## Ethics Statement

The studies involving human participants were reviewed and approved by Scientific and Research Ethics Committee of the Medical Research Council, Budapest, Hungary. The patients/participants provided their written informed consent to participate in this study.

## Author Contributions

XG, DG, DB, GJ, and GB: conceptualization. DB, NE, SS, and PP: methodology. XG, NE, DB, DT, and ZG: data collection. DG, ZK, SS, PP, DB, NE, DT, and ZG: data analysis. XG, DG, SS, ZK, ZG, DT, and DB: writing—original draft preparation. XG, DG, NE, ZG, DT, DB, ZK, SS, PP, GJ, and GB: writing—review and editing. All authors contributed to the article and approved the submitted version.

## Conflict of Interest

The authors declare that the research was conducted in the absence of any commercial or financial relationships that could be construed as a potential conflict of interest.

## Publisher's Note

All claims expressed in this article are solely those of the authors and do not necessarily represent those of their affiliated organizations, or those of the publisher, the editors and the reviewers. Any product that may be evaluated in this article, or claim that may be made by its manufacturer, is not guaranteed or endorsed by the publisher.

## Supplementary Material

Supplementary Table 1 Main effect of *CLOCK* SNPs in the NewMood database surviving quality control on lifetime depression (DEP). Results of logistic regression models and quality control steps are shown. While there were nominally significant SNPs, significant clumps could not be identified.

Supplementary Table 2 Main effect of *CLOCK* SNPs in the NewMood database surviving quality control on current depressive symptoms (BSI-DEP). Results of linear regression models and quality control steps are shown. While there were nominally significant SNPs, significant clumps could not be identified.

Supplementary Table 3 *CLOCK* SNPs in the NewMood database surviving quality control, in interaction with recent life events (RLE) on current depressive symptoms (BSI-DEP). Results of linear regression models and quality control steps are shown. SNPs in significant clumps are marked in red, top SNP of the clump is marked in bold red. Results for additive, dominant, and recessive models are shown.

Supplementary Table 4 *CLOCK* SNPs in the NewMood database surviving quality control, in interaction with childhood adversities (CHA) on current depressive symptoms (BSI-DEP). Results of linear regression models and quality control steps are shown. SNPs in significant clumps are marked in red, top SNP of the clump is marked in bold red. Results for additive, dominant, and recessive models are shown.

Supplementary Table 5 Main effect of *CLOCK* SNPs in the NewMood database surviving quality control on recent negative life events (RLE). Results of linear regression models are shown. There were no nominally significant SNPs, significant clumps could not be identified.

Supplementary Table 6 Main effect of *CLOCK* SNPs in the NewMood database surviving quality control on childhood adversities (CHA). Results of linear regression models are shown. There were no nominally significant SNPs, significant clumps could not be identified.

Supplementary Table 7 *In silico* characterization and functional prediction of SNPs in the clumps significantly interacting with recent life events (RLE) and childhood adversities (CHA). Top SNP of each clump is marked in bold red.
